# Digital medication and patients' right of autonomy in Spain

**DOI:** 10.1111/bioe.13335

**Published:** 2024-07-30

**Authors:** Salvador Pérez Álvarez

**Affiliations:** ^1^ Faculty of Law, Open University of Spain (UNED) Universidad Nacional de Educacion a Distancia Madrid Spain

**Keywords:** adherence, digital pill, health data, patient autonomy, right of autonomy

## Abstract

The progress the Internet has experienced in recent years has brought about huge changes and social transformation in all aspects of our lives. One such aspect greatly impacted has been our health, where we can talk about the existence of an ‘Internet of Medical Things’. Amid this digital drift, we have seen the development of pharmaceutical drugs that provide information to patients and their attending healthcare teams concerning medication, doses ingested, and time of ingestion. These are digital pills or digital medication. In this context, the purpose of my paper is to analyze the ethical and legal impact of digital medication, further analyzing the implications concerning the right of service users to make decisions over their own health in Spain.

## 1 INTRODUCTION

The progress the Internet has experienced in recent years has brought about huge changes and social transformation in all aspects of our lives. Even the transition from Web 1.0 to Web 2.0 contributed to a true Cyberculture,^1^ thanks to the complex system of business operations and social interactions that were more and more frequently conducted over the Internet.^2^ This version of the Internet was based on transfer and communication protocols such as the hypertext transfer protocol and the simple mail transfer protocol for connecting applications. However, today's online environment and its architecture enables the exchange of goods and services amongst all elements, equipment, and objects connected to cyberspace. This is how what we call Internet of the Things (IoT)^3^ is born.

The IoT refers to the interrelation of everyday objects that are often equipped with some type of artificial intelligence (AI)‐enabled devices. AI is a technology trying to emulate the human brain's multiple abilities to produce intelligent behaviours, synthesizing and automating cognitive tasks typical of the human consciousness.^4^ Those systems fitted with such intelligence require a sequence of instructions or algorithms that determine the actions of objects or whichever other technology. The introduction of sequential algorithms to the IoT has allowed cyberspace to become a platform for devices that communicate electronically and share specific data and information with their surrounding environment.^5^ This is true for every setting where we currently make increasingly more frequent use of such technologies. One of such settings is health care, where we can speak of the existence of an Internet of Medical Things (IoMT) or ‘healthcare IoT’^6^ that, based on Morla's submission ‘refers to those medical devices connected to the Internet with the purpose of better addressing the user's needs who at the same time (in this case) is a patient’.^7^

At the beginning of the 21st century, we saw the advent of the first few technological systems used to establish degrees of medication adherence by patients,^8^ automatic pill dispensing devices, mobile phone applications reminding patients when they must take their medication, and even containers with sensors that record whenever medication has been taken out. Amid this new digital drift, the company Proteus Digital Health, Inc., patented an ingestible sensor that could be embedded into a tablet for oral administration. Once in contact with stomach acids, the sensor sends a signal to a patch, which would then inform patients and their attending healthcare professionals about which drug has been taken, the dose, and time of ingestion. The aim of such a product was to improve the efficiency of medication intake following the dose required to deal with the illness being treated and at the same time reduce the risks associated with excessive intake.^9^ In 2012, the United States Food and Drugs Administration (FDA) authorized the combined use of this sensor with other pharmaceutical products.

Proteus started collaborating with different pharmaceutical companies to develop drugs with such a system. And out of this collaboration, AbilifyMyCite was developed: a combined system with aripiprazole (an atypical antipsychotic) and a sensor, the use of which was approved by the FDA in November 2017 for the treatment of schizophrenia, manic episodes, and mixed episodes associated with bipolar disorders and depression in adults.^10^ This biotechnological breakthrough was the prelude to a new generation of drugs, digital medication. Digital medication was shaped to be a new source of information, the accuracy, and objectivity of which would allow the development of personalized therapies for each individual patient.^11^ The flipside of such developments with the ever‐more‐usual ‘smart’ drugs is that we must carefully consider the implications of so much innovation for the exercise of self‐determination by users of this type of medical products. Given these assumptions, the aim of this paper is to analyze the influence of digital medication over the autonomy of underaged patients under Spain's current legal system.

## 2 RIGHT TO CHOOSE MEDICAL CARE AND DIGITAL MEDICATION

Based on Tarado Socia's proposition, self‐determination in relation to one's own health is one of the manifestations of the autonomy of health service users, which consists of the freedom to make any healthcare‐related decision following the moral imperatives of our (freedom) of conscience.^12^ We are all entitled to our own opinions and points of view and to make our own decisions in relation to any matter pertaining to our health based on our values and/or beliefs.^13^ In Spain's healthcare setting, the valid exercise of personal autonomy in this respect involves, *inter alia*, the freedom to choose any available therapeutic option and the right to data protection of the user's medical records.^14^ The foregoing is in accordance with sections 2.3 and 7.1 of the Spanish Act 41/2002, of 14 of November, Minimum Implementing Regulations on Patient Autonomy and the Rights and Obligations concerning Clinical Information and Documentation [*Act 41/2002*]. Further, it is precisely the prescription and uptake of digital medication that directly influence such manifestations of self‐determination concerning one's own health.^15^

### 2.1 Influence of digital medication on the right to decide over intake and level of adherence to treatment

#### 2.1.1 A patient's right to freely decide on adherence

Prescribing medication is the professional medical act to avail a patient of a treatment for an illness or condition they are suffering. Pursuant to the provisions of Section 40.3.i of the Spanish Act 44/2003, of 21 November, on the Management of Healthcare Professions (the MHP Act) [*Act 44/2003*], healthcare professionals have the freedom to prescribe ‘having regard to the requirements of scientific knowledge’. That is to say, the capacity to choose the most convenient treatment among available drugs (complying with safety and efficacy criteria) so that the patient may cope with the symptoms they are suffering.^16^ Accordingly, the Supreme Court leaves little room for doubt when determining that ‘the physician, in the course of his professional practice, is free to choose the most beneficial solution for the patient's welfare, making the resources they deem most effective available to the case under review, as long as said resources are generally accepted by medical science or subject to scientific debate, and having regard to those risks attached to the medical act in practice, to the extent of the physician's commitment to their obligation of finding the means to reach a diagnosis and adequate medical therapy aimed at preserving life, human integrity and patient health’.^17^

The medical profession's freedom to prescribe must be exercised to serve society, for the benefit and in favour of the health of the patient. Further, such freedom must strictly comply with the obligations arising from the physician's professional code of ethics (Section 4.3 of the MHP Act). In 2022, Spain's General Medical Council enacted their most recent Code of Medical Ethics and Professional Conduct (CEDM).^18^ Article 10 of the updated Code makes a provision for the exercise of said freedom: that the physician must respect the patient's freedom to decide on therapy options and the patient's freedom to reject such options (whether wholly or in part), respect the freedom to undergo a diagnostic test, or even to adhere to a specific treatment. Therefore, the patient's right to self‐determination in matters of health care in accordance with the moral imperatives of their (freedom) of conscience limits the prescription liberties of healthcare practitioners. Although, the extent of such limitation on the freedom of medical prescription does vary depending on whether we are dealing with scenarios of voluntary, aesthetic, or curative interventions.^19^ In the first instance, it is the patient who voluntarily commissions medical services and could request of the medical professionals the use of a specific medical procedure or drug. While in the case of curative interventions, the patient may refuse to take the medication in whole or in part at their own risk, they cannot demand a specific prescription of the physician in contravention of the *lex artis* or against the physician's medical knowledge (based on current science) in relation to the patient's condition.^20^

If a patient freely chooses to take a medication prescribed for curative purposes, the physician's freedom to prescribe includes the authority to monitor, inter alia, the level of adherence on the basis of such prescription.^21^ That is, the patient's adherence to therapy.^22^ Adherence entails that the patient must take the medication subject to the dose prescribed for as long as indicated.^23^ For such adherence, it is necessary that the physician shares the instructions of use and intake of a prescription in writing beforehand with the patient, clearly and in a manner accessible and adequate to the patient's needs (Section 4.2 of the Spanish Act 41/2002). Healthcare professionals support that proper patient information is vital for maintaining a good patient–doctor rapport, which means the patient must be given all the necessary information. If we explain the reasons and purpose behind the prescription given, the patient will understand and accept any decisions made concerning their health more willingly, favoring in turn better adherence to any such treatment.^24^ And likewise the patient must inform their physician regarding the level of medication intake in accordance with the dose and frequency prescribed so that the attending physician is able to monitor two things: efficacy of the drug used to prevent or cure the patient's condition and compliance with the terms of the treatment course prescribed.^25^

Notwithstanding the foregoing, the patient is not legally obligated to adhere to treatment in the terms prescribed nor to inform on the level of adherence to treatment.^26^ The World Health Organization (WHO) makes it very clear that adherence requires the patient's freely and voluntarily given consent with regard to the recommended use and intake of the drug indicated by the attending healthcare team.^27^ In this respect, authorities (doctrine) emphasize that nonadherence to treatment by patients is influenced by social, medical, or psychological factors and, as such, adherence may vary throughout the period of illness and also depending on the type of illness.^28^ Then, it follows that adherence to a specific drug is one of the manifestations of a patient's right to choose adherence or noncompliance with a course of treatment as prescribed or even if at some point they stop such course of treatment relying on their personal autonomy in matters of health and moral imperatives. Adherence is also related to personal beliefs concerning the need to take medication, the fear of possible side effects that treatment adherence may entail.^29^

The valid exercise of this ‘right to decide on adherence to medication’ comes with a caveat when dealing with patients whose capacity to follow medical guidelines on their own is hindered. I am referring to very elderly patients or patients with cognitive impairment due to neurodegenerative disease.^30^ If we are talking about users legally incapacitated (or evaluated by the attending physician as de facto incompetent) to make reasoned medical decisions, the level of prescription supervision ought to be in my view carried out by the legal representative, as the person who consented to and accepted the therapeutic option prescribed on behalf of the former (Section 9.3 of the Spanish Act 41/2002). The legal representative must also inform the attending physician about adherence to the prescribed course of medication on behalf of the person they represent (the principal). In this way, the physician can supervise if the treatment is effectively preventing or curing the condition. However, any such intervention by the representative must be appropriate and proportionate to the relevant needs and always for the health benefit of the principal and having regard to the service user's personal dignity. The incapacitated service user must still be engaged in any decisions pertaining to the prescribed treatment's adherence to any extent possible (Section 9.5 of the Spanish Act 41/2002). In this regard, the Preamble of the UN Convention on the Rights of Persons with Disabilities of 13 of December 2006 (CRPD) stresses the importance of ‘individual autonomy and independence, including the freedom to make their own choices’, which includes health‐related decisions.

The exercise of the right to choose on matters of treatment adherence is more complex in the case of patients who are ‘capable’ in principle but who are sufferers of mental health disorders such as schizophrenia, bipolar disorders, and other mental health conditions. Adequate medication is fundamental to reduce the risk of adverse outcomes in those cases, such as psychosis, recurrence of symptoms, social dysfunction, hospitalizations, and suicide attempts.^31^ Something similar occurs with very elderly patients, who despite having legal capacity (to make medical decisions)^32^ have difficulties following a course of prescribed medication because of age‐related diminished cognitive capacity.^33^ Given those scenarios, the work of some authorities (doctrine) has revealed that adherence to treatment increases in quantitative terms when monitored by a de facto carer.^34^ In these cases, it would be desirable that the carer could take on the responsibility of informing the attending physician about the patient's compliance, if and only if the patient consents. Where possible, the patient must also be involved in any decisions related to treatment adherence and supervision.

#### 2.1.2 Ethical and legal aspects of the right to choose on matters of adherence to digital medication

When searching for a method to improve rates of treatment adherence, the main obstacle up to now has been the almost exclusive reliance of healthcare professionals on the information provided by patients. Failing that, on the information provided by legal representatives or de facto carers. Nevertheless, the information provided either by the service user (or their representatives on their behalf) is not always reliable. This is because of the patient's inability to follow guidelines properly or because they choose not to inform on treatment adherence or lack thereof, asserting their personal autonomy.^35^ If we wish to remedy the deficiencies arising from lack of adherence, perhaps digital medication could be an extremely useful tool to minimize the risks of non‐adherence to treatments as prescribed. Digital medication could also help attending healthcare teams to monitor drug efficacy.^36^ Since the FDA approved the use of AbilifyMyCite in 2017 to the present time and above all as a result of the impact of the COVID‐19 pandemic on our current health systems, IQVIA has made it known that several companies in different countries have registered around 250 patents for the development of digital medication to combat different types of disease.^37^ There were 25 official authorizations for prescription purposes to treat diabetes, AIDS, tuberculosis and various other mental health illnesses, cardiovascular disease, and cancer.^38^

These new generation drugs make adherence to the prescribed treatment easier for patients. In the case of some digital medication, the encapsulated sensor swallowed is connected to an external patch that allows data collection concerning treatment efficacy. This information is sent to an application installed on the patient's mobile phone and to any relatives that would have previously consented to receiving such data. Further, the information reaches a portal where the patient's medical record is stored and handled by the attending physician. Said mobile application generates alerts and reminders, etc., for the patient to know when they need to take the medication, change the patch, or have their mobile device at hand to keep on funnelling their treatment adherence data.^39^ Other digital pills have been developed to release their medication based on the information provided by the sensor. This eliminates the need of drug intake at the times specified in the prescription, as this happens automatically.^40^ Some other digital drugs take images ‘from the inside’ showing the areas of the body affected by the disease in question, which are then sent to an external mobile device.^41^

Such digital medication makes treatment adherence easier particularly for elderly patients or patients with neurodegenerative conditions who can then follow the course of treatment as prescribed. At the same time, it affords the attending physician a better and more reliable control of treatment adherence^42^ because it makes monitoring possible on an ongoing and immediate basis. Thus, the physician keeps on receiving the most relevant patient health data throughout the course of the treatment.^43^ During the 24th National Psychiatry Congress and in reference to mental illnesses such as schizophrenia, bipolar, and depressive disorders, Barrigón concluded that digital devices allow for very useful screening, especially for planning the provision of mental healthcare services to patients suffering from those conditions.^44^ The use of such digitalized medication makes for a possible and considerable amortization of the resources allocated to public healthcare services, which translates into a reduction of the cost arising from bad treatment adherence.^45^ However, the use of digital medication raises a number of legal‐ontological quandaries in relation to the full enjoyment of service users' right to choose on actual and effective equal terms.

First, not all citizens have equal access to those drugs. To begin with, the use of digital medication has only been approved in countries such as the United States. In contrast, the European Medicines Agency for instance has yet to approve their distribution and commercialization within the European Union.^46^ But we must remember that even in countries where the use of digital medication has been authorized, there is an accessibility gap because the cost of such medication is far higher than any equivalent generic version.^47^ This means that digital medication is only accessible to those users with sufficient purchasing power in contrast to others who have the same conditions but cannot afford digital medication.^48^ This is why currently only a small percentage of all potential users around the world is able to enjoy the advantages of adhering to digital medication to benefit their health to the detriment of others who cannot because of where they live or cannot afford such medication.^49^ With a view to lessening discrimination in terms of access to digital medication, it would be desirable for the relevant health authorities in WHO member countries to authorize the distribution and use of those digital drugs compliant with pharmacological safety protocols to the effect. Once authorized globally, it would also be necessary for health authorities to subsidize access to digital medication under medical prescription. At least, I would argue, in favour of those users who are unable to afford such medication by themselves.

Second, the use of medication with encapsulated sensors such as AbilifyMyCite (which collects and transmits treatment adherence data to an application installed on an external telephonic device) involves the use of modern smartphones with Bluetooth and an internet connection. Despite the wide range of fully enabled affordable devices currently in the market to be used by all people in combination with such technologies, not all users avail themselves of such applications for their everyday lives. They would all refuse (in exercise of their right to choose on health matters) to subject themselves to digitally medicated treatments involving the use of such technologies that ensure treatment adherence in terms of efficacy and effectiveness.

Section 81 of the Spanish Constitutional Act 3/2018, of 5 December, on Personal Data Protection (DP) [*Act 3/2018*], sets out that ‘all citizens are entitled to internet access regardless of personal, social, economic or geographic status’. However, a few decades ago the Special Senate Committee on Computer Networks set forth that utilization of technologies in Cyberspace is a free and voluntary act and never mandatory.^50^ Such freedom includes the right not to be treated with drugs developed through the IoT, based on the individual's ideology, thinking, or beliefs that may be contrary to the use of application or tools created in said version of the Web. The Spanish Constitutional Court has made it clear that when a user employs such applications they are ‘revealing information about their personality, which may affect the deepest core of their intimacy [conscience] as it relates to ideologies, religious beliefs, hobbies, health information, sexual orientation, etc.’^51^

Naturally, among those who do not wish to use (or refuse the use of) such technological advances, we find patients who would benefit the most from a treatment adherence programme prescribed in the form of digital medication. I am referring to very elderly users and those with serious mental health conditions.^52^ In relation to the former (although many have been avid users of such technology for over a decade^53^), there is a wide range of mental health patients who do not have the skills to use applications similar to those that must be installed on a mobile phone for the management of information received via digital pills.^54^ In fact, the Spanish Ministry for Work and Social Economy in its National Accessibility Plan (2004–2012) already contemplated the question of impeded accessibility to state‐of‐the‐art mobile phones by the visually impaired or people with learning disabilities, in relation to their skills and ability to handle such devices on their own.^55^ We observe a similar picture with very elderly patients, who generally speaking tend to least adhere to prescribed courses of treatment for reasons beyond their free and consciously consented will, as stated by the WHO. The Spanish Intergenerational Solidarity Association announced that in 2022 the rate of smartphone use in our country by people of 50–60 years of age was 65%. This percentage falls to 22.95% in the case of users over 80.^56^ Spain's Office for National Statistics (INE) has reported that only 27.8% of smartphone users over 65 know how to set up and use the applications installed on their phones by themselves.^57^

People with mental health conditions or the very elderly are not freely and consciously refusing to use digital medication, particularly in the knowledge of how beneficial such medication can be for their health as a higher degree of adherence is ensured.^58^ In most cases, their refusal to ingest such modern treatments is actually due to their lack of skill in handling mobile phone applications for the purposes under discussion. Digital wisdom can be defined as ‘the ability to find practical, creative, contextually appropriate, and emotionally satisfying solutions to complicated human problems’^59^ through the applications and tools of the IoT. Digital wisdom thus defined is an attribute of the human conscience. The development of this empowers individuals to have access to systems of participation and communication in this version of the Web. Such access would be essential for the exercise of citizenship competence in an ever more digitalized society.^60^

To close the digital gap that separates those patients who would benefit the most from these new generation drugs, it is necessary that public bodies put educational measures in place for the use of applications to manage adherence to prescribed courses of treatment.^61^ The authorities ought to implement such measures to guarantee universal right of access to the Internet, as provided for under Section 81 of the Spanish DP Act. In accordance with the provisions of Section 81.4 of said Act, it is public authorities who must guarantee and, where applicable, eliminate any obstacles to the valid exercise of this right through measures aimed at training older people on the use of technologies typical of the information society. If we are dealing with patients with learning difficulties or impairments, those measures must be rolled out as part of any holistic care schemes developed by the relevant health authorities for the benefit of those patients. This is the only way in which the patients could decide freely and voluntarily if they wish to be treated with digital medication and wish to adhere to such treatments through mobile devices, based on their own moral (freedom) of conscience. Then, there would be no need to have this decision made by their carers or legal representatives.

Lastly, digital medication directly impacts patient's autonomy in relation to making decisions that could affect their health throughout the course of treatment. From the outset, we encounter two issues. One is that the patient or, if need be, the patient's legal representative must consent freely and voluntarily to the ingestion of such type of drugs when prescribed by their attending physician. And further we observe that they must accept specific conditions of use when installing sensor‐data‐collecting applications and when setting up the medical device. This is usually done with nonnegotiable, standard‐form contracts that require the acceptance of all contract clauses before proceeding.^62^ The problem lies with the way such consent is informed. Ordinarily, the physician would inform the patient clearly and in writing about the treatment and any associated risks, paying attention to the treatment's peculiarities. However, in most cases standard‐form contracts (in the context of information society services) are draughted in general terms and with rather complex jargon by the very companies providing those services. This means that most potential users of digital pills find such wording hardly intelligible, not to mention elderly users or mental health service users.^63^ If we consider that the attending physician is the prescriber of such applications, should it not be the physician's role to inform patients clearly about the terms of use of such medication? In this way, the user can choose freely and voluntary whether to submit to such therapy option or not.^64^ And in the case of legally incapacitated patients, the information must be provided to their legal representative, who will have to agree to the terms and conditions of use of the application linked to the digital pill on behalf of the patient. The representative will always have to act for the health of the principal, who must participate to the extent possible in the decision of whether to accept such terms and conditions of use or not.

Digital medication that releases the treatment dose, limits patient's autonomy, the freedom to choose treatment adherence or interruption, based on the imperatives of their own (freedom of) conscience or, even worse, because of side effects. In this respect, some cases have been identified where the pill or drug continued to release its medication causing cardiac arrhythmia, worsening the cardiovascular health of the patient (IQVIA, 2021). If we want to ensure the right to choose in matters of health in such scenarios, it is necessary that the service user (for the benefit of their own health) or the relevant representative, whether legal or de facto, can stop the drug release through the mobile device application that receives the sensor's data. Further, in any case, the user should be able to stop treatment at any point through their device, therefore exercising their right to self‐determination in accordance with the moral imperatives of their own (freedom) of conscience.^65^

Lastly, digital medication has a direct impact on or influence over the patient's right to decide concerning prescribed treatment course adherence or treatment interruption. As we have been saying so far, the development of such drugs mainly seeks treatment adherence, as per the course of treatment prescribed by the patient's attending physician. Supervision of treatment adherence is carried out thanks to the information provided by the digital pill's sensor or the patch to which the sensor is connected. Data are collected about actual ingestion, dose of ingestion, and time of ingestion by the patient. However, during the treatment, the patient is free to decide whether to inform the physician if they have complied with the prescribed course or not. According to the WHO, there is a quantitative drop in the level of adherence when dealing with chronic diseases and treatments are long term. Further, there is such a drop in patients from developing countries whose resources are limited and cannot afford the full treatment or part of it. Treatment adherence is also related to personal beliefs and the need to continue taking prescribed medication when faced with the potential side effects that may arise from drug intake.^66^ This is usually the case with prescribed cancer medication because the side effects are very invasive for the health of cancer sufferers.^67^

Notwithstanding the moral responsibility arising from patient's nonadherence to a prescribed treatment based on freedom of choice,^68^ it is also true that patients can remove the patch that receives sensor information (or not renew it at expiration in the case of such devices) if they do not wish to continue providing information to their doctor, based on personal autonomy and in accord with the moral imperatives of their own (freedom) of conscience. For more advanced digital medication that works through embedded sensors providing health data, the patient should be given the option to control the flow of information through the application installed on their mobile device linked to the pill.^69^ If not table to do it by themselves or through the legal representative acting on their behalf, as the case may be, we would be facing an unjustified limitation of patient autonomy concerning their reporting of treatment adherence. Even further, in Spain, the intake of such medication could be injurious to the patient's ‘right to confidentiality concerning their health‐related data and their right to no third‐party access to such information without prior authorization as protected by Law’ (Section 7.1 of the Spanish Act 41/2002). Let us examine this issue in more detail.

### 2.2 Data protection for health data processed via digital medication

The information compiled through digital medication concerning the efficacy and adherence of prescribed treatments are data pertaining to someone's own health. That is to say, ‘personal data pertaining to the physical or mental health of a natural person, including healthcare service provision data that may reveal information about the person's health status’ in accordance with the provisions of Article 4, Regulation (EU) 2016/679 of the European Parliament and of the Council of 27 April 2016 on the protection of natural persons (General Data Protection Regulation, GDPR). Under EU law and under Spanish law, said data are construed as a special category of personal data, and the processing thereof must follow the assurances contemplated by the GDPR and the Spanish DP Act; for the sake of safeguarding the right to privacy of users of such new generation drugs.^70^

Subject to the approach taken by the Spanish Data Protection Agency (Agencia Española de Protección de Datos, AEPD), attending medical teams can have access to adherence information concerning patients undergoing digital medication treatments (who have freely and voluntarily consented to treatment) without the need for permission to process their health data and in accordance with the provisions of Article 6.1.c) of the GDPR.^71^ The data controller will be the health centre holding the patient's medical records (Section 34.1 of the Spanish DP Act). The health centre in question will include the information on treatment adherence relying on the data provided by the digital medical device. Such data access shall be restricted exclusively to the user's biometric information as strictly necessary to assess the efficacy and level of adherence to treatment, which is the purpose of such data collection.^72^ Further, those data will become part of the patient's clinical records (Section 15 of the Spanish Act 41/2002), and only the healthcare practitioners directly involved in treating the patient can access the information (and any other professional consulted by the attending team for the purposes of improving the patient's therapeutic care).^73^

Digital medication provides medical teams (and others as expressly authorized by the patient) with treatment adherence information and other biometric data generated by the patient.^74^ Biometric data are data obtained from specific processing (in this case, digital medication) relating to the physical, physiological, or behavioural characteristics of the user of such type of medication (Article 4 of the GDPR). Some of the biometric information collected by the digital device is strictly necessary to supervise the patient's level of adherence to the drug. However, the healthcare professionals should not have access to behavioural data related to the patient's everyday habits if such data bears no relation to the medical treatment in question, unless the patient may have consented to this freely and expressly (articles 4 & 9.2 of the GDPR), and provided that, relying on the approach of the AEPD, such information is strictly necessary for the purposes of the medical treatment for which the data have been yielded.^75^

Even if the attending health team can have access to all data pertaining to the health of the digital medication user (data provided by the digital device without the need for patient consent subject to current data protection), the patient may at some point freely and voluntarily decide to stop providing such health information. This being the case, the patient, or the patient's legal representative, can exercise their right to object to further processing of their data being received by the medical team from the digital pill (Section 18 of the Spanish DP Act and articles 21–22 of the GDPR). The exercise of the right to object to data processing is an expression of patient autonomy^76^ and is part of the patient's right to decide over their own health and in accord with their (freedom) of conscience. These data are being stored electronically by the attending healthcare centre, which means that by exercising said right of objection the user of digital medication is availing themselves of a constitutional guarantee on the right to digital freedom or *habeas data*. According to Spanish Constitutional Court doctrine, [such constitutional remedy] ‘includes an authority over the use and control of personal data that (…) legally expressed [in statue] in the power to consent to compilation, collection and access to personal data, subsequent storage and processing of personal data, as well as its use or possible uses by a third party, whether a state or an individual’.^77^ Except for reasons of public interest such as control of communicable diseases, epidemics, cross‐border threats, and so on,^78^ the patient cannot object to the healthcare practitioners accessing the data provided by the digital medication device (Article 9.2.i of the GDPR).

Beyond such cases, when using digital medication together with external patches for gathering treatment adherence information, the patient or the patient's legal representative must inform the attending physician of the intention to exercise the right to object to further provision of such health data. The removal of the patch is what would be simply required to exercise such right or not replacing the patch when the current one has expired. Then, there will no further information transmission to the computer programme used by the healthcare team attending the user, subject to the imperatives of their own (freedom) of conscience. When using more advanced technology, treatment adherence information is provided by the sensor embedded in the digital pill. In this case, the patient's right to objection should be exercisable through the application installed on the mobile phone and linked to the device in question. The application should allow the patient to cancel the flow of data being sent to the computer programme used by the healthcare team. Said computer programme is where all health and/or biometric data are recorded for monitoring adherence to the prescribed treatment.^79^

Healthcare professionals with access to such patient data (obtained thanks to new generation drugs) are subject to confidentiality rules once the information has gone into the patient's medical records and in accordance with the provisions of Section 7.1 of the Spanish Act 41/2002. As noticed by Morla, ‘under the umbrella of confidentiality we therefore find other roles subject to keeping patient health data confidential such as administrative staff, documentalist, IT personnel, etc.’^80^ This also includes the companies that have developed the digital medication and the computer programmes supporting such medication. According to the AEPD, the standard‐form contract that the patient or the patient's legal representative, as the case may be, must sign to be able to ingest the digital pill acts as legal cover so that companies can access those data that are strictly necessary to fulfil the purpose of these medical treatments,^81^ that is, to assess treatment efficacy and patient adherence to the prescribed course of therapy. In any event, the patient or the patient's legal representative should be duly informed about the processing of any personal data obtained thanks to these biotechnologies together with the identity and contact details of the company's controller accessing the health‐related information in real time and the purposes of the processing intended for such personal data (Article 13.1 of the GDPR).

In general, the companies that develop such medication and technologies need the data to conduct research protocols that allow them to review the effectiveness of their products, whether to enhance them or to develop other more advanced products.^82^ Therein lies the problem. Unless there are reasons of public interest to the contrary, the data from digital medication devices may be used for research, provided that they comply with the Seventh Additional Provision of the Spanish DP Act as follows: (i) that the service user (or their legally authorized representative) has thus expressly consented to it; (ii) that biometric data identifying the person are separated (as the data cannot be used for personal identification purposes) from clinical care data; (iii) that the data may have been previously pseudonymised with an express undertaking to keep them confidential; and (iv) that specific security measures are taken to prevent personal identification of the data subject. In any case and in accordance with the Spanish Constitutional Court's opinion, the patient has the right to object at any moment to the company's continued use of their personal data gathered from the digital pill for the purposes of scientific research.^83^ In my view, full enjoyment of the right to object acts as guarantor for the patient's right to choose adherence to a digital medication treatment in accordance with the imperatives of their freedom of conscience.

## 3 CONCLUDING REMARKS

I. One of the main problems faced by public health services nowadays is the lack of treatment adherence by patients subject to the terms of the prescription given by their attending teams. As the WHO has pointed out, lack of adherence to medical treatment usually happens in patients with chronic conditions, even more in very elderly patients or patients with mental health disorders or neurodegenerative disorders that affect volition and cognition. Unless contraindicated, incorrect ingestion of recommended and prescribed doses of medication has a negative effect on patients' health and considerably increases healthcare services costs because we are subsidizing treatments that are not adhered to wholly or in part by service users.
II. When trying to find a method for improving adherence rates up to now, healthcare professionals have relied on the information provided by patients almost exclusively. This information was not reliable, whether because treatment guidelines were not being followed properly or because of the decision of not informing on treatment adherence concerning the prescribed course of therapy (in the exercise of personal autonomy). With a view to combating nonadherence deficiencies in this second decade of the 21st century, pharmaceutical companies from different countries have developed the so‐called digital medication or digital pills. Proteus Digital Health patented an ingestible sensor that could be embedded in a pill to be taken orally. The sensor would send a signal to a patch after coming into contact with stomach acids, sending information to patients and to their physicians about the drug, dose, and time of ingestion. In 2017, AbilifyMyCite was developed and authorized to market in the United States to treat schizophrenia, manic episodes, and mixed episodes associated with bipolar disorder and adult depression. Since then, other similar drugs that facilitate treatment adherence in the manner prescribed have been patented. Some of them have an encapsulated sensor ingested by patients connected to an external patch that allows for data collection concerning treatment efficacy. The information is sent to a specific smartphone application on the patient's phone, even to the patient's relatives' phones (if patients have previously consented to this). This information is also sent to the portal containing the patient's medical records handled by the attending physician. Currently, other pills that release the medication into the patient based on information from the sensor have been developed so that it is no longer necessary for patients to manually ingest anything as there is an automatic scheduled release of medication.
III. If the use of such medication has been approved by the relevant health authority, healthcare professionals are entirely free to prescribe these drugs (if they deem such treatment is the most appropriate to prevent or cure a certain condition in one of their patients), based on scientific rigour and after weighing the benefit‐risk of such use. However, the physician must respect patient autonomy in compliance with the Code of Medical Ethics and Professional Conduct and when exercising the physician's freedom to prescribe. That means observing the patient's freedom to choose nonadherence to the treatment as prescribed in whole or in part. The patient has the right to decide if they consent to digital medication therapies to treat their condition. They also have the right to decide over adherence to treatment in the manner prescribed and based on the information provided by the digital pill, which is a specific manifestation of their personal autonomy in everything related to their own health and in accordance with the imperatives of their own (freedom) of conscience.
IV. The information provided to the attending physician by the digital medication about treatment efficacy and patient adherence to therapy is personal data related to the patient's own health and part of the patient's medical records. According to the approach by the Spanish Data Protection Agency, the attending healthcare team may have access to adherence data in relation the treatment that the patient has freely and voluntarily consented to but without having to seek specific consent to process the patient's own health data. Nevertheless, healthcare teams may have access to other biometric data through these digital therapies for which express consent must be sought, unless those data are necessary to monitor the level of medication efficacy in relation to the condition in question. In any event, the user has the right to object to the attending healthcare team continuing to process the health data obtained via digital medication. The exercise of this right of objection to treatment processing of health data is an expression of autonomy by users of digital medical therapies, which in my view acts as an institutional assurance of the patient's right to decide over adherence to digital therapy, subject to the imperatives of their own (freedom) of conscience.

## Footnotes

1 Levy, P. (2001). *Cyberculture.* University of Minnesota Press.

2 As stated by Castells: ‘Once in place as powerful technology embedded in social practice, the Internet, has a rather relevant impact on innovation and further on the development of new forms of culture, broadly and narrowly. That is, in terms of being an integral part of social thinking and in terms of cultural and artistic creation, respectively.’ Castells, M. (2003). La dimensión cultural de Internet. *Andalucía Educativa*, *36*, 8.

3 Salazar, J., & Silvestre, S. (2017). *Internet de las cosas*. TechPedia (European Virtual Learning Platform for Electrical and Information Engineering).

4 The origin of AI dates from a theoretical computer science conference in 1956 at Dartmouth College in the United States, where scientists Allen Newell and Herbert Simon introduced *Logic Theorist*, a computer programme that emulated human brain functions. This was considered the first AI system as it was able to prove mathematical logic theorems. Navas Navarro, S. (2017). Derecho e inteligencia artificial desde el diseño. Approximations. In S. Navas Navarro (Eds.), *Inteligencia artificial, tecnología y Derecho* (Tirant Lo Blanch).

5 Salazar and Silvestre, op. cit., note 4.

6 De Miguel Beriain, I. (2022). Desarrollo de mecanismos de inteligencia artificial por parte de las entidades de salud. Seis consejos para evitar problemas éticos y jurídicos. In Cotino Huseo, L., & Todolí Signes, A. *Explotación y regulación del uso del big data e inteligencia artificial para los servicios públicos y la ciudad inteligente*. Tirant Lo Blanch.

7 Morla González, M. (2019). Medicamentos digitales. La autonomía del paciente a debate. *Revista Internacional de Eticas Aplicadas*, *29*, 121.

8 Adherence to medication has been defined by authorities as the patient's compliance with their treatment in the terms prescribed by the patient's physician. Vrijens, B. De Geest, S., Hughes, D. A., Przemyslaw, K., Demonceau, J., Ruppar, T., Dobbels, F., Fargher, E., Morrison, V., Lewek, P., Matyjaszczyk, M., Mshelia, C., Clyne, W., Aronson, J. K., Urquhart, J., & ABC Project Team. (2012). A new taxonomy for describing and defining adherence to medications. *British Journal of Clinical Pharmacology*, *73*(5).

9 Egilman, A. C., & Ross, J. S. (2019). Digital medicine systems: An evergreening strategy or an advance in medication management? Evidence‐Based Medicine, *24*(6).

10 Press release of 13 November 2017. https://www.fda.gov/news-events/press-announcements/fda-approves-pill-sensor-digitally-tracks-if-patients-have-ingested-their-medication

11 Morla González, op. cit., note 7.

12 Tarodo Soria, S. (2005). *Libertad de conciencia y derechos del usuario de los servicios sanitarios*. Servicio Editorial de la Universidad del País Vasco.

13 Sánchez‐Caro J., & Abellán, F. (2000). *El consentimiento informado*. Fundación Salud. Thus understood, the right to decide over one's own health is a realization of the health service users' freedom of conscience implicitly enshrined under Article 16.1 of the Spanish Constitution, which includes the right to one's ideas, opinions, or beliefs, to express them, to behave in accordance with such opinions, ideas, or beliefs and not to be forced to go against them. Llamazares Fernández, D. (2011). *El derecho de la libertad de conciencia I. Conciencia, tolerancia y laicidad*. Civitas‐Thomson Reuters.

14 The confidentiality of the data within medical records is protected, among other rights and freedoms, by the right to decide over one's own health, insofar as it forms part of the patient's most intimate space; respect for which is essential for the patient to be able to make any decisions they deem most appropriate concerning their well‐being, without any external interference other than that necessary for the attending healthcare team to be able to inform them of their state of health and of the medical treatments the patient may undergo on the basis of their free choice or the medicines that may be prescribed to treat the illness in question. Tarodo Soria, op. cit., note 12.

15 Morla González, op. cit., note 7.

16 Sánchez‐Caro, J., & Abellán, F. *Derechos del médico en la relación clínica* (Fundación Salud 2000, 2006).

17 Spanish Supreme Court Decision 363/2006, 8 February, Legal Foundation 3.

18 Retrieved from: INT DEONTOLOGÍA 13×21 03_2023.indd (aeds.org).

19 The purpose of curative medicine is to heal a patient presenting a pathological alteration of their physiology. On the other hand, voluntary medicine acts on a healthy body to improve its aesthetic appearance, such as cosmetic, perfective, or beautifying surgery, or to remove its reproductive capacity. Palma de Mallorca's Supreme Court Ruling 155/2014, 18 June, Legal Foundation 3).

20 Spanish Supreme Court Decisions 1159/1996, 23 February, Legal Foundation 1; 8562/1999, 30 December, Legal Foundation 3; 822/2018, 3 January, Legal Foundation 2.

21 The doctor is, therefore, ‘in charge of indicating the individualized therapeutic treatment according to the patient's response and of prescribing the use or intake of medication and the supervision thereof, providing adequate information concerning its use’. Spanish Constitutional Court Decision 363/2006, 8 February, Legal Foundation 3.

22 The WHO has defined adherence as ‘the degree to which a person's behaviour—taking medication, following a diet and making lifestyle changes—corresponds to the agreed recommendations of a healthcare provider’. Yach, D., et al. (2004). *Adherencia a los tratamientos a largo plazo. Pruebas para la acción.* WHO, Chapter 1 (1).

23 Castro, A., et al. (2010). *La adherencia al tratamiento: Cumplimiento y constancia para mejorar la calidad de vida*. Pfizer. http://envejecimiento.csic.es/documentacion/biblioteca/registro.htm?iPos=11&id=57526&irPag=1&clave=xu5H8qi7iJ&pos=0

24 ibid.

25 Morla González, op. cit., note 7.

26 Larios Risco, D. (2011). Libertad de conciencia y prescripción de medicamentos. Extensión y límites. In M. Sánchez (Eds.), *Libertad de conciencia y medicamento*. Comares.

27 Yach, op. cit*.*, note 22.

28 Castro, A., et al., op. cit., note 23.

29 According to the WHO, the degree of treatment adherence decreases quantitatively in the case of chronic diseases or in the case of patients in developing countries where health resources are very few. Yach, op. cit., note 22.

30 Morla González, op. cit., note 7.

31 Litvinova, O., Klager, E., Tzvetkov, N. T., Kimberger, O., Kletecka‐Pulker, M., Willschke, H., & Atanasov, A. G. (2022). Digital pills with ingestible sensors: Patent landscape analysis. *Pharmaceuticals*, *15*, 1025.

32 According to the Spanish Act 8/2021, of 2 of June, on the Protection of Persons with Disabilities.

33 Morla González, M. (2020). El control de la adherencia en el tratamiento de personas mayores a través del empleo de medicamentos digitales. In E. Atienza Macias (Ed.), *Soluciones tecnológicas para los problemas ligados al envejecimiento: cuestiones éticas y jurídicas*. Dykinson.

34 Vicente‐Sánchez, S. et al. (2018). Adherencia al tratamiento en pacientes mayores de 65 años que sufren reingresos precoces. *Farmacia Hospitalaria*, 42(4).

35 Morla González, op. cit., note 32.

36 De Miguel Beriain, I., & Morla González, M. (2020). Digital pills for mental diseases: An ethical and social analysis of the issues behind the concept. *Journal of Law and Biosciences*, *7*(I).

37 ‘Digital health had been slowly becoming part of the therapeutic paradigm alongside traditional medicines before COVID‐19, and this process has been somewhat accelerated by regulatory agencies adapting to unprecedented times’. IQVIA, ‘Institute report on digital health trends 2021. Innovation, evidence, regulation, and adoption’ (2021), p. 4. Retrieved from: https://www.iqvia.com/insights/the-iqvia-institute/reports/digital-health-trends-2021

38 Litvinova et. al., op. cit., note 31.

39 Morla González, op. cit., note 7.

40 The use of this type of medication has the potential advantage of reducing the possible anxiety that may arise for the patient's next of kin if they do not adhere to the treatment as prescribed by their doctor. De Miguel Beriain & Morla González, op. cit., note 35.

41 Litvinova et. al., op. cit., note 31.

42 Siddesh Shewalkar, K., et al. (2021). Digital pills: Impact of rising technology. *Archives of Medicine* 13(6).

43 De Miguel Beriain and Morla González, op. cit., note 35.

44 ML Barrigón, M. L. (2021). Uso de smartphones para el screening poblacional en salud mental. *Paper presented at the XXIV Spanish National Conference of Psychiatry*. http://psiqu.com/2-66113

45 Morla González, op. cit., note 32.

46 Martani, A., et al. (2020). Digital pills: A scoping review of the empirical literature and analysis of the ethical aspects. *BMC Medical Ethics*, *21*(3).

47 Therefore, for instance, the cost of AbilifyMyCite treatment was over $1600 per month in the United States compared to $500 or $600 per month with the generic Abilify*.* Litvinova, O., et al., op. cit., note 31.

48 Siddesh Shewalkar, op. cit., note 41.

49 De Miguel Beriain and Morla González, op. cit., note 35.

50 The Special Senate Committee on Computer Networks think that ‘all persons have the fundamental right of free access to the Internet regardless of sex, status, appearance, mental characteristics, age or place of residence (…) There must be full freedom of access, distribution, information and communication’ (N. 4.2). On the other hand, the exercise of this right entails the freedom not to access services that are typical of the information society, and the freedom not be forced to.

51 Spanish Constitutional Court Decision 173/2011, 7 November, Legal Foundation 3.

52 Morla González, op. cit., note 7.

53 Fernández Rodríguez, J. (2013). Integración social de los menores con discapacidad a través de Internet. In S. Pérez Álvarez (Ed.), *Menores e Internet*. Thomson Reuters, Aranzadi.

54 Barrigón, op. cit., note 43.

55 Retrieved from: https://www.mdsocialesa2030.gob.es/derechos-sociales/discapacidad/informacion/index.htm

56 Retrieved from: https://solidaridadintergeneracional.es/wp/los-smartphones-todavia-no-seducen-a-los-mayores-en-espana/

57 INE. (2021). Encuesta sobre equipamiento y uso de tecnologías de información y comunicación en los hogares. https://www.ine.es/jaxiT3/Datos.htm?t=46292

58 Morla González, M. (2024). *Medicina personalizada de precisión y Derechos Fundamentales*. Tirant Lo Blanch.

59 Presnky, M. (2010). Homo Sapiens digital: De los inmigrantes y nativos digitales a la sabiduría digital. In R. Aparici (Coord.) *Conectados en el ciberespacio*. Servicio de Publicaciones de la UNED, p. 96.

60 Pérez Álvarez, *S.* (2020). *Libertad de conciencia y diversidad en la sanidad pública española contemporánea*. Tirant Lo Blanch.

61 Morla González, op. cit., note 57.

62 Morla González, op. cit., note 7.

63 Fernández Rodríguez, op. cit., note 52.

64 In this respect, the Constitutional Court of Spain has made it clear that ‘for this power to consent (to decide on the medical acts affecting the subject) to be exercised in full freedom, it is essential that the patient is in possession of adequate medical information on the therapeutic measures, since only if they have this information can they freely give consent, choosing between the options presented to them; or decide, also in full freedom, not to authorize the treatments or interventions proposed by the doctors. In this way, consent and information are manifested as two rights so closely intertwined that the exercise of one depends on having previously tended correctly to the other, which is why the unjustified deprivation of information is equivalent to the limitation or deprivation of the very right to decide and consent to medical action, thus affecting the right to physical integrity of which this consent is a manifestation’. Spanish Constitutional Court Decision 37/2011, 28 March, Legal Foundation 5.

65 De Miguel Beriain and Morla González, op. cit., note 35.

66 Yach, op. cit., note 22.

67 Garfield, S., et al. (2011). Suitability of measures of self‐reported medication adherence for routine clinical use: A systematic review. *BMC Medical Research Methodology*, *11*.

68 Litvinova, O., et al., op. cit., note 31.

69 Fleisch, E., et al. (2021). *The digital pill.* Emerald Publishing.

70 Personal health data are protected by the right to privacy under Article 18 of the Spanish Constitution in accordance with the Constitutional Tribunal authority. Spanish Constitutional Court Decision 232/1993, 12 July, Legal Foundation 3.

71 AEPD. (2022). *Guía para profesionales del sector sanitario*. https://www.aepd.es/es/documento/guia-profesionales-sector-sanitario.pdf

72 Tarodo Soria, op. cit., note 12.

73 AEPD, op. cit., note 69.

74 Morla González, op. cit., note 57.

75 AEPD, op. cit., note 69.

76 Fleisch, S., et al., op. cit., note 67.

77 Spanish Constitutional Court Decision 17/2013, 31 January, Legal Foundation 4.

78 AEPD, op. cit., note 69.

79 Fleisch et al., op. cit., note 67.

80 Morla González, op. cit., note 7, p. 127.

81 AEPD, op. cit., note 69.

82 Morla González, op. cit., note 7.

83 The power to avail themselves of these data is a manifestation of the digital medicine user's right to digital freedom that includes ‘the right to control the use of the data within a computer programme (*habeas data*) and it includes, inter alia, the citizen's objection to the use of certain personal data for purposes other than the intended purpose declared by the data collector’. Spanish Constitutional Court Decisions 290/2000, 30 November, Legal Foundation 7; 96/2012, 7 May, Legal Foundation 6; 17/2013, 31 January, Legal Foundation 4; 198/2012, 6 November, Legal Foundation 7; 58/2018, 4 June, Legal Foundation 5; 164/2021, 4 October, Legal Foundation 3; 23/2022, 21 February, Legal Foundation 3.

